# *Equus caballus* Papillomavirus Type-9 (EcPV9): First Detection in Asymptomatic Italian Horses

**DOI:** 10.3390/v14092050

**Published:** 2022-09-15

**Authors:** Livia De Paolis, Chiara Grazia De Ciucis, Simone Peletto, Katia Cappelli, Samanta Mecocci, Tiziana Nervo, Lisa Guardone, Maria Ines Crescio, Daniele Pietrucci, Floriana Fruscione, Federica Gabbianelli, Silvia Turco, Katia Varello, Gian Guido Donato, Cristiana Maurella, Paola Modesto, Maria Grazia Maniaci, Giovanni Chillemi, Alessandro Ghelardi, Elisabetta Razzuoli

**Affiliations:** 1 National Reference Center of Veterinary and Comparative Oncology (CEROVEC), Istituto Zooprofilattico Sperimentale del Piemonte, Liguria e Valle d’Aosta, Piazza Borgo Pila 29/34, 16129 Genova, Italy; 2 S.C. Diagnostica Specialistica, Istituto Zooprofilattico Sperimentale del Piemonte, Liguria e Valle d’Aosta, Via Bologna, 148, 10154 Torino, Italy; 3 Dipartimento di Medicina Veterinaria, Università degli Studi di Perugia, Via San Costanzo 4, 06126 Perugia, Italy; 4 Dipartimento di Medicina Veterinaria, Università degli Studi di Torino, Largo Paolo Braccini 2, Grugliasco, 10095 Torino, Italy; 5 S.C. Osservatorio Epidemiologico, S.S. Biostatistica, Epidemiologia e Analisi del Rischio, Istituto Zooprofilattico Sperimentale del Piemonte, Liguria e Valle d’Aosta, Via Bologna, 148, 10154 Torino, Italy; 6 Dipartimento per la Innovazione nei Sistemi Biologici, Agroalimentari e Forestali (DIBAF), Università degli Studi della Tuscia, via San Camillo de Lellis snc, 01100 Viterbo, Italy; 7 Dipartimento di Scienze Agrarie e Forestali (DAFNE), Università degli Studi della Tuscia, via San Camillo de Lellis snc, 01100 Viterbo, Italy; 8 Azienda USL Toscana Nord-Ovest UOC Ostetricia e Ginecologia, Nuovo Ospedale Apuane, Via Enrico Mattei 21, 54100 Massa, Italy

**Keywords:** EcPV9, virus detection, gene expression, Italy, horse, fertility

## Abstract

Papillomavirus (PV) infections may be related to anogenital lesions and cancer development in humans and several other animal species. To date, 11 different PVs have been reported in horses. Among them, a newly described PV named *Equus caballus* Papillomavirus Type9 (EcPV9) was thus far only reported in the semen of a stallion with penile lesions in Australia. This study reports for the first time the presence of EcPV9 in asymptomatic Italian horses. From July 2020 to January 2022, genital brush samples were collected from 209 horses with no apparent signs of neoplastic disease and no PV-associated lesions, clinically examined at the Didactic Veterinary University Hospital (OVUD) of Perugia and at the Veterinary University Hospital (OVU) of Turin. Brushes were submitted to real-time PCR targeting the EcPV9-*L1* region. The first amplification targeted a region of ~116 bp, followed by the amplification and sequencing of ~533 bp of the positive samples. EcPV9-*L1* DNA was found in eleven horses (5.3%), all female and mainly English Thoroughbred. Co-infection with EcPV2-*L1* was found in 7 out of the 11 EcPV9-*L1* positive horses (63.6%). This study contributes to the description of the prevalence of exposure or infection of EcPVs in the horse population in Italy, for which data are still limited. In this regard, here we provide a phylogenetic analysis and the completely reconstructed viral genomes of two Italian EcPV type 9 isolates, as well as four EcPV type 2 obtained from co-infected animals.

## 1. Introduction

Papillomaviruses (PVs) are a group of highly host-adapted, small, non-enveloped DNA viruses. They show a specific tropism for cutaneous and mucosal keratinocytes, and infection is generally thought to require epithelial wounding or micro-wounding to allow access of the virus to the basal lamina [1,2,3]. In the last decades, the PV group has received great attention due to its connection with cancer development and its wide distribution throughout vertebrates [4]. Both benign and malignant lesions have been attributed to PVs in many animal species, including humans [1,4,5,6]. To date, more than 200 human papillomavirus (HPV) genotypes have been reported [5,7]. Among these, HPVs classified as low risk, such as HPV type 6 and 11 [8], typically cause asymptomatic/subclinical infections, or papillomas, which are generally resolved by the host’s immune system after months or years [2]. However, other HPV types are associated with cervical, anogenital, and head and neck squamous cell carcinomas (HNSCC) [1]. For instance, HPV types 16 and 18 are the two most common genotypes found in cervical lesions, causing 60–80% of all genital cancers [9]. Moreover, pharynx cancers are increasingly attributed to infection with HPV, primarily HPV16 [10], and are thus considered high-risk HPV (hrHPV) [11,12]. Although the role of PVs in tumor development and progression still needs to be cleared, viral integration in the host genome is generally associated with disease severity. Integration causes loss or disruption of the early E2 gene encoding for regulatory proteins, which in turn causes dysregulation of E6 and E7 oncogenes, determining their increased expression and enhanced production of related oncoproteins [2,13]. Genomic features and the etiological significance of anogenital-associated PVs in animals are not as well characterized as in humans, and the association between some PV type(s) and disease phenotype(s) is still uncertain [5]. Nevertheless, PVs from different types of anogenital lesions have been reported in non-human primates, cetaceans, domestic animals, rodents and bats [5,6,14,15,16,17,18,19].

As regards Equidae, to date, 13 PVs have been documented to infect horses and donkeys: *Bos taurus* papillomavirus 1 (BPV1), 2 (BPV2), and 13 (BPV13), *Equus caballus* papillomaviruses 1–8 (EcPV1–8), *Equus asinus* papillomaviruses 1–2 (EaPV1–2) [20]. In addition, a newly described equine PV named EcPV9 was reported in the semen of a stallion with penile lesions in Australia, through the characterization of the complete 7605 bp genome sequence and phylogenetic analysis based on concatenated E1-E2-L2-L1 amino acid sequences [20]. Equids PVs are classified on the basis of nucleotide sequence diversity in the late L1 gene, which is the most conserved PV gene and, thus, the most frequently used for detection and molecular identification.

Several studies provided evidence for an active involvement of PV infection for cancer development also in horses [13,21,22]. In particular, EcPV2 was found in gastric, penile, vulvar, clitoral and oro-pharyngeal papillomas, as well as in both in situ (CIS) and invasive squamous cell carcinomas (SCCs) [13,21,22], and EcPV8 was found to be associated with viral plaques, viral papillomas, and SCCs [23].

In this study, we describe for the first time the presence of EcPV9 in Italian horses, and we provide the complete genome reconstructed from two different animals. Phylogenetic analyses were performed to compare these EcPV9 Italian isolates with the other ones available in the public databases, confirming their relationships with the EcPV9 reported in the semen of a thoroughbred stallion in Australia.

## 2. Materials and Methods

### 2.1. Genital Brush Samples Collection from Horses for EcPV9 Detection

From July 2020 to January 2022, genital brush samples were collected from horses during routine clinical examination at the Didactic Veterinary University Hospital (OVUD) of Perugia and at the Veterinary University Hospital (OVU) of Turin for medical reasons unrelated to the study, with the following inclusion criteria: (i) no apparent sign of neoplastic disease; (ii) no PVs associated lesions. No restrictions were set for breed, age and sex.

Penile and vulvar swabs were collected through sampling with sterile cytobrushes (Deltalab SLU, Barcelona, Spain). Vulvar swabs were taken from the vaginal vestibulum of mares, while penile swabs were obtained from stallions and geldings by gently rubbing the glans mucosa, close to fossa glandis.

The brushes were stored in 2 mL tubes containing 800 µL of DNA/RNA Shield Stabilization Solution (Zymo Research, Irvine, CA, USA), then kept at −20 °C until processing.

### 2.2. DNA Extraction and Real-Time PCR Targeting EcPV9-L1 and EcPV2-L1

DNA extraction was carried out using a QIAamp DNA Mini Kit (QIAGEN, Milan, Italy), according to manufacturer’s instruction. For each sample, total DNA was extracted from 200 µL of DNA/RNA Shield Stabilization Solution (Zymo Research, Irvine, CA, USA) in which cytobrushes were previously kept. Samples were eluted in 100 µL of elution buffer (QIAGEN, Milano, Italy), and DNA was quantified by Qubit fluorimeter (Thermo Fisher Scientific, Waltham, MA, USA). EcPV9-*L1* detection was assessed in 100 ng DNA samples by using primers and related specific probes for the amplification of a target region of ~116 bp (Table 1); equine beta-2-microglobulin (β2M) gene primers previously described (Table 1) were used for testing DNA amplifiability. Primers and probes sequences are reported in Table 1. Internal controls (block blanks, extraction blanks and positive controls) were used for each analytical session. A field sample previously confirmed by Sanger analysis was used as positive control. Real-time PCR was performed in a CFX96 Real-Time System (BioRad, Milan, Italy) by using iTAQ universal probes supermix (BioRad, Milan, Italy), 200 nM of the probe, 100 nM of each primer; for each sample, 100 ng/5 µL were added to 20 µL of the reaction mix. A threshold cycle of 38 was set as cut-off for virus positivity. For assessing the specificity of EcPV9-*L1* primers, amplification products were sequenced using PCR primers on a 3130XL Genetic Analyzer (Thermo Fisher Scientific Inc., Waltham, MA, USA). Sequences were aligned using the SeqMan software (Lasergene package. DNASTAR Inc., Madison, WI, USA) to obtain a consensus sequence and compared with available sequences retrieved from the National Center for Biotechnology Information (NCBI) database through the BLAST tool.

EcPV2-*L1* presence was also assessed for EcPV9-*L1* positive samples, by following the previously described protocol. Primers and related specific probes used for EcPV2-*L1* detection are reported in Table 1.

### 2.3. L1 Sequencing Results and Molecular/Phylogenetic Analysis

A fragment of ~533 bp was targeted for the EcPV9-*L1* sequencing in positive samples. The amplification was obtained through Platinum™ Taq DNA Polymerase (Thermo Fisher Scientific, Carlsbad, CA, USA) using the primer pairs reported in Table 1. Standard conditions for the PCR reactions were: 10× PCR buffer, 5 μL of DNA, 1.25 μL 20 μM of each primer, 1.25 μL 10 mM of dNTPs, 1.25 units of Platinum^®^ Taq DNA Polymerase, 1.25 μL of 50 mM MgCl_2_ in a final volume of 25 μL. Thermocycling parameters consisted of an initial denaturation step (94 °C, 2 min) followed by 40 cycles of denaturation (94 °C, 30 s), annealing (60 °C, 30 s) and extension (72 °C, 1 min), amplifications were run on a CFX 9600 (BioRad, Milan, Italy). The PCR products were separated on 2% agarose gels, the bands were excised from the gel and purified using the NucleoSpin^®^ Gel and PCR Clean-up (Macherey-Nagel, Düren, Germany). Purified DNA was used for sequencing of both strands with the Big Dye Terminator v.1.1 cycle sequencing kit (Applied Biosystems, Massachusetts, Waltham, MA, USA). The consensus sequence was determined by the alignment of forward and reverse strand using SeqMan (Lasergene package, DNASTAR Inc., Madison, WI, USA). The consensus sequences were compared by Blast analyses with the only EcPV9 sequence deposited in GenBank. A multiple sequence alignment including the newly generated L1 sequences and sequences from other EcPVs was built with BioEdit v.7.0.5.2 using CLUSTALW [24]. Nucleotide substitution models were evaluated using jModelTest2 [25], and the best model was selected according to the Akaike information criterion (AIC) analysis. MEGA7 was used for phylogeny inference according to the Maximum Likelihood (ML) criterion. The nucleotide substitution model was set according to jModelTest2 output and was Kimura-2 with a Gamma distribution and Invariant sites (GTR + G + I). The robustness of the hypothesis was tested in 1000 non-parametric bootstrap iterations.

**Table 1:** Primers set and probes for EcPV9-*L1* detection and sequencing and for EcPV2-*L1* detection.

Gene	Sequences	Amplicon Length	Accession	Reference
EcPV9-*L1*	F-5′- TTC ATC CCA GCT TGA GAC CA-3′	116	MN117918.1	This paper
	R-5′-GCA GAT CAA TGG TCC AGA AGG -3′			
EcPV9-*L1* seq	F-5′-AGG AGA TGT ATG TTG CCC GT -3′	533		
p-EcPV9-*L1*	p-FAM- ATT GCC TCC TCA GCC ACC CG-TAMRA			
*EcPV2-L1*	F-5′-TTGTCCAGGAGAGGGGTTAG-3′	80	NC_012123	[26]
	R-5′-TGCCTTCCTTTTCTTGGTGG-3′			
*p-EcPV2-L1*	p-FAM-CGTCCAGCACCTTCGACCACCA-TAMRA			
*B2M*	F-5′-CTGATGTTCTCCAGGTGTTCC-3′	114	NM_001082502.3	[26]
	R-5′-TCAATCTCAGGCGGATGGAA-3′			
p-*B2M*	p-FAM-ACTCACGTCACCCAGCAGAGA-TAMRA			

### 2.4. NGS and Bioinformatics Analysis

We carried out an NGS-based identification of the EcPV9 genome sequence for the samples where enough DNA was available. In detail, total DNA was extracted from five horse samples listed in Table S3 and sequenced at the Institute of Applied Genomics (IGA, Udine, Italy) using paired-end sequencing methods on an Illumina NovaSeq 6000 machine. The quality of the raw reads was then evaluated by FastQC [27] and the Illumina adapters trimmed with Trimmomatic v0.39 [28], using the following parameters: LEADING:15 TRAILING:15 SLIDINGWINDOW:4:15 MINLEN:111 HEADCROP:11.

To keep only the non-horse reads, the *Equus caballus genome* (EquCab2.0, GCF_000002305.2) was used as reference in a filtering step performed using BWA v 0.7.12 aligner and samtools v1.13 [29,30]. For a de novo assembly of the filtered reads, SPAdes v3.15.4 [31] was used with default parameters, and the resulting assembled contigs were blasted toward an in-house built database including the EcPV complete genomes available on the National Center for Biotechnology Information (NCBI) database (Table 2). The contigs related to EcPV were manually processed, when needed, to reconstruct the complete viral genome, which was further validated by alignment of the filtered raw reads using BWA. Samtools was used to calculate the coverage and the Integrative Genomics Viewer [32] for alignment visualization. The coding sequences of the reconstructed viral genomes were identified using the Genome Annotation Transfer Utility (GATU) [33]. A phylogenetic analysis was performed by aligning the reconstructed sequences to the EcPV genomes of the database using MUSCLE [34], to build a maximum likelihood (ML) tree with RaxML-HPC, using the GTRCATI algorithm as substitution model and 1000 bootstraps [35]. The tree was then visualized in a dendrogram using FigTree v1.4.4 (http://tree.bio.ed.ac.uk/software/figtree, accessed on 22 June 2022).

### 2.5. Statistical Analysis

According to submission information, horses were classified by age as “young” (<8 years) or “adult” (≥8 years). Breed and origin information was obtained from medical records. Microsoft Excel (2016) software was used for descriptive statistics analysis such as mean ± 1 standard deviation and median calculations of age, male and female, and proportion of positivity. Moreover, STATA16.1 (StataCorp, College Station, TX, USA) software was used to fit a logistic regression model assessing the association, expressed as odds ratio (OR) between the positivity or negativity to EcPV9-*L1* (dependent variable) and 4 classes of age (group 1: <6 yy; group 2: 6–<9 yy; group 3: 9–<13 yy; group 4: ≥13 yy) and 2 of breed (English Thoroughbred vs. the others) (independent variable). Statistical analysis was then restricted to the mares: the OR was assessed through logistic regression using as dependent variables the positivity/negativity to EcPV9-*L1*, artificial insemination/natural service, pluriparous/maiden, while breed and age were considered as independent variables. Finally, a third logistic regression model was fit to assess the OR between being pregnant vs. being empty (dependent variable) and the positivity/negativity to EcPV9-*L1* and fertility/ipofertility (independent variables).

## 3. Results

### 3.1. Sampled Horses

A total of 209 horses (195 females, 11 stallions and 3 geldings) were sampled in this study during clinical examination at OVUD (Perugia) and OVU (Turin). The age ranged from 6 months to 24 years, with a mean of 10.2 (±5.2 SD) years and a median of 10 years. Overall, 31.1% of sampled animals were <8 years old (young) and 65.6% ≥8 years old (adult). The age was not known for seven animals. Moreover, following the four categories of age division, 41 animals were <6 yy (very young), 34 of 6–<9 yy (young), 62 of 9–<13 yy (adult) and 65 of ≥13 yy (elderly). The sampled animals belonged to different breeds: 89 Italian Standard Breed (42.6%), 72 English Thoroughbred (34.4%), 12 Italian Saddle (5.7%), 9 Arabian Thoroughbred (4.3%), 4 Quarter Horse (1.9%), 4 Shire (1.9%), 3 Belgian (1.4%), 2 Hannover (1%), 2 Maremman (1%), 2 Anglo Arabian (1%), 2 ponies (1%), 1 Sardinian Anglo Arabian (0.5%), 1 Appaloosa (0.5%) and 1 Argentine Criollo (0.5%). Moreover, the breed was not known for five horses (2.4%). Sampled animals came from various Italian regions: 128 (61.2%) were from Piedmont, 31 (14.8%) from Umbria, 13 (6.2%) from Lazio, 9 (4.3%) from Tuscany, 8 (3.8%) from Emilia-Romagna, 5 (2.4%) from Marche and Lombardy, 3 (1.4%) from Sardinia, 2 (1%) from Campania, 1 (0.5%) from Sicily and France, while for 3 (1.4%), the geographical origin was unknown.

### 3.2. Detection of EcPV9-L1 and EcPV2-L1

Overall, EcPV9-*L1* DNA was found in 5.3% (11 out of 209) of the examined horses. These animals were subjected to clinical examination.

Moreover, among the eleven horses positive for EcPV9-*L1* DNA, seven of them (63.6%) also showed the presence of EcPV2-*L1* (Table S1). All of the positive subjects were mares. The age of the positive subjects ranged from 8 to 15 years of age, although the age was not known for two subjects. The geographical origin was: Piedmont (5); Sicily (1); Tuscany (1); Emilia Romagna (2) and Umbria (2) (Figure S1). As regards the breed, horses positive for EcPV9-*L1* DNA were all English Thoroughbred except for one Shire horse (Table 3). The logistic regression model showed that English Thoroughbred had an OR of 26.4 (IC 95% 3.2–214.7) to result positive for EcPV9 with respect to other breeds used as comparison variable.

Regarding the age, a higher percentage of positive subjects was observed in group 3 (9.7%) compared to group 2 (5.9%) and 4 (1.6%), while no positive subjects were found in group 1 (Table 4). The age was not known for 2 of the 11 positive samples. No significant associations with positivity to EcPV9 were seen on the basis of the age.

### 3.3. Detection of EcPV9 in Mares

Among the 209 horses enrolled in this study, 158 were broodmares, which belonged to different breeds: 87 (55.1%) were Italian Trotter, 62 (39.2%) English Thoroughbred, 4 (2.5%) Shire, 2 (1.3%) Arabian Thoroughbred and Italian Saddle. Moreover, it was not possible to know the breed of one horse. Their age ranged between 3 and 21 years, with an average of 10.4 (±4.4 SD) years and a median of 10 years. English Thoroughbred and Shire were subjected to natural service, whereas Italian Trotter, Italian Saddle and Arabian Thoroughbred to artificial insemination. Among the sampled mares, 119 were pluriparous and 28 maiden. It was not possible to obtain this information for 11 mares. As mentioned in Section 3.2, all 11 horses positive for EcPV9 were mares; thus, in this group, the prevalence was 7% (11 out 158), including 10 English Thoroughbreds and 1 Shire horse (Table 5). In this group of animals, the logistic regression model showed that English Thoroughbred had an OR of 18.1 (IC95% 2.2–145.2) to be positive to EcPV9 with respect to other breeds used as comparison variable. Overall, the 8.4% of pluriparous (10 out 119) resulted as positive to EcPV9. It was not possible to obtain data on the number of births given for one of the positive mares. No significant difference was found on EcPV9 genoprevalence between maiden and pluriparous. As regards the breeding type, 18% (11 out 61) of mares subjected to natural service were positive to EcPV9, while no mares subjected to artificial insemination were found positive. No association was found between the positivity or negativity to EcPV9-*L1* and artificial insemination vs. natural service. No significant association was found nor between positivity/negativity to EcPV9 and fertility/hypofertility, neither between positivity/negativity to EcPV9 and being pregnant/being empty. Moreover, all positive mares were followed by a veterinarian until March 2022. No signals of PV-related lesions were detected. However, seven animals out nine showed reproductive problems during follow-up (Table S2).

### 3.4. L1 Sequencing Results and Molecular/Phylogenetic Analysis

EcPV9-*L1* sequences of 533 bp were obtained for all eleven positive samples. The quality of the sequences was checked manually using Sequencing Analysis v. 5.2 (Applied Biosystems). The sequences were then used for the alignment of the forward and reverse sequences of each sample in order to obtain a consensus sequence for phylogenetic analysis.

**Figure 1:**
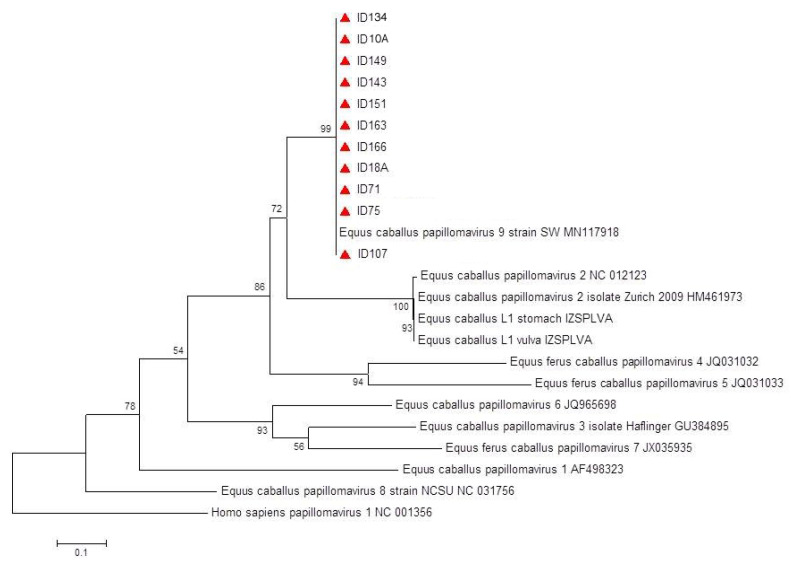
Phylogenetic tree. Phylogeny was inferred by maximum likelihood (ML) analysis obtained by an alignment of 533 bp of the L1 gene of EcPV. EcPV type and GenBank accession numbers are indicated. Sequences generated in this study are indicated by a red triangle. Bootstraps (1000 replicates) values >50 are shown at the internal nodes. The length of each pair of branches represents the distance between sequence pairs. The scale bar represents the percentage of nucleotide differences.

All nucleotide sequences obtained were identical to each other and showed a 100% similarity to EcPV9 strain SW (MN117918.1). A 405-nucleotide alignment including the newly generated sequences, other EcPV sequences, and human PV1 as outgroup, was used for phylogenetic analysis. The phylogenetic tree confirmed that sequences identified in this study cluster with EcPV9 within the Dyoiota genus (Figure 1).

### 3.5. NGS and Bioinformatic Analysis

The total number of raw reads obtained from Illumina sequencing, together with the number of trimmed and filtered reads, is reported in Table S3. The presence of EcPV9 in the assembled contigs was evaluated with Blast, and the results are shown in Table S4. In detail, in samples ID75 and ID107, only EcPV9 was detected, with the former showing one single contig (NODE_2, length 7758 bp) with the complete EcPV9 genome, and the latter showing more contigs that were manually curated in order to reconstruct the complete genome (Table S4). In samples ID71, ID125, and ID166, the presence of contigs related to both EcPV2 and EcPV9 further supported the double-infection information obtained from the real-time PCRs. Note that we were able to reconstruct the complete viral genome for all co-infected EcPV2 samples, with the exception of ID125, where an almost complete one was nevertheless obtained.

The completely reconstructed sequences, either related of EcPV2 or EcPV9, were further validated by mapping the raw reads with BWA, calculating the breadth and depth coverage with samtools (Table S3) and visualizing the alignment with IGV (Figure S2). Once validated, the sequences were deposited, together with their annotated features, under the accession numbers (ON989000–ON989004).

The phylogenetic relationship among the newly reconstructed EcPV isolates is in line with their papillomavirus type (Figure S3): as expected, the three novel EcPV2 isolates clustered among the type 2, while the two novel EcPV9 isolates clustered with the type 9 strain SW. When aligned, the two new EcPV9 isolates resulted to be almost identical to the SW strain, showing only three SNPs along the genome (positions 899, 1189 and 2196), two of which were undetermined based on the SW reference and the third one on a repeated region rich of guanine bases (Figure S4).

## 4. Discussion

This study represents the first investigation on EcPV9 genoprevalence in horses worldwide and contributes to the description of EcPVs epidemiology in Italy, a viral infection for which data are still limited [36,37]. It also represents the first report of EcPV9 in the country, validated by molecular characterization of *L1*, phylogenetic analysis and viral genome reconstruction by NGS.

As concerns the molecular characterization, PV genes are grouped into early (E) and late (L), depending on their expression phase during the infection. Among late PV genes, the *L1* is the most conserved and is thus widely used for PV detection and identification by sequencing. Moreover, after the infection, the viral genome can be integrated into the host DNA or maintained as multiple episomes that replicate concomitantly to the host cells [2]. Viral integration has been associated with disease severity, since the integration of viral DNA into the host genome causes loss or disruption of the early E2 gene. In turn, E2 loss causes dysregulation of E6 and E7 oncogenes, determining their increased expression and the consequent enhanced production of related oncoproteins [2,3].

In this study, the full genome of two EcPV9 was obtained; thus, we can speculate that in these two animals, the viral genome was not integrated into the host genome. The role of this new virus in EcPV-related lesions and its possible involvement in cancer will need to be further investigated. In humans, to date, over 200 types of HPVs have been characterized. However, the majority are classified as “low-risk” (lrHPV) types, and about 12 types as “high risk” oncogenic PVs (hrHPV16, 18, 31, 33, 35, 39, 45, 51, 52, 56, 58, 59) [38]; more specifically, HPV16 and HPV18 are involved in 70% of cases of cervical cancer, including high-grade squamous intraepithelial lesions [38,39]. As it occurs in humans, also in horses, different PV infections are associated with different lesions; in particular, EcPV1, 3–7 have been associated with aural and genital plaques [40,41], EcPV4 with aural CCS [23], and EcPV1, 8 with cutaneous papillomas and papillomatosis [42,43]. Moreover, EcPV2 was found in penile, vulvar, clitoral and oro-pharyngeal papillomas, as well as in both CIS and invasive SCCs [22,44]. SCC is the most common malignant cutaneous tumor in horses, developing mostly on non-pigmented skin and muco-cutaneous areas. In particular, the external genitalia, as well as the ocular region, are reported to be the most commonly SCCs-affected areas [45,46]. In veterinary literature, the association of equine vulvar SCCs with PVs has been reported in few cases [22], but the real prevalence is still unknown. However, to the authors’ best knowledge, all published cases were positive to EcPV2 [21,36,44,47,48,49]. In a previously described clinical case involving an Italian pony, multifocal areas of squamous epithelial hyperplasia associated with moderate nucleocytoplasmic atypia, together with an in situ carcinoma (vulvar intraepithelial lesion, VIN), were considered precursors of SCC, as hypothesized in women [36]. SCC can also develop in the stomach, being the most common primary equine gastric tumor, (3–4% of all equine SCCs) [22,50]. Thus far, only a recent case series on the possible viral etiology of equine gastric carcinomas has been reported [36,47,48].

Concerning EcPV9, as mentioned, it was thus far only detected in the semen of a Thoroughbred stallion with a penile lesion in Australia [20]. In the present study, at variance, all positive horses were female, presenting no clinical symptoms or evident lesions. As no biopsy samples could be taken from the stallion of the Australian case, the authors state that it was not possible to confidently determine the significance of the viral infection in relation to the observed pathologies. Nevertheless, the novel EcPV described was extracted from semen samples, collected when a wart-like lesion was visible on the tip of the penis, and hence compatible with a disease syndrome caused by a papillomavirus, also considering that no other microbial pathogens were detected by meta-transcriptomic analysis [20]. In the present study, virus occurrence was observed in 11 healthy subjects. In seven of them (63.3%), EcPV9 was found in association with EcPV2, a major etiologic agent of equine SCC disease, suggesting that EcPV9 could be associated with papillomavirus-related malignancies in horses. This observation needs to be further confirmed in future studies; however, it shows a condition of co-presence already observed in horses with BPV1 and BPV2 and clinically evident in human HPV related pathologies [51]. In this respect, only about 20 PV types can be associated with HPV-related cancers. Moreover, HPV detection is not necessarily indicative of disease, considering that in about 90% of the cases, viral clearance occurs and women with cervical intraepithelial neoplasia grade 1 (CIN1) show about 70%-90% of regression rate, respectively, within 1 and 2 years after diagnosis [52]. Concerning EcPV2 infection in horses, a recent study demonstrated a high genoprevalence and low incidence of EcPV2 related lesions; thus, viral clearance can be speculated in horses such as in humans [37]. Further studies should highlight this aspect in relation to EcPV9.

The sequences of EcPV9 isolated in this study differ from the Australian sequences only for three SNPs. This suggests higher conservation of this type of EcPV compared to EcPV2 where even isolates from the same country differ among each other. Among HPV types, sequence diversity can be found, where intratype variant lineages differ by 1–10%, and sublineages by 0.5–1%. Since PVs are double-stranded DNA viruses, they can use their host proofreading DNA polymerase for their replication, thus avoiding high mutation rates. For this reason, mutations in HPV genomes have been acquired slowly, defining HPV types whose infection cycle has adapted to different host cells. The nucleotide polymorphisms of PV intratype variants derives from random mutations occurring within viral types generated [53].

Our findings further prove the broad diversity of PV types in horses and highlight the need for further investigation of the clinical significance of this newly described virus. The clinical impact of the nine EcPVs affecting the equine species is still not well characterized. In addition, there is increasing evidence for the involvement of PVs in the development of canine, ovine and feline SCCs [54,55,56,57,58]. Similarly, it is accepted today that human infection by specific HPV types chiefly contributes to SCC development and maintenance. There is evidence for 90% of diagnosed cervical cancers, 50% of anogenital tumors and 22% of head and neck SCCs being caused by hrHPV types [59,60,61], mainly HPV16 and HPV 18 [7]. HPV oncoproteins E6 and E7 have been recognized as essential factors in HPV-induced carcinogenesis [59].

Our results show that in horses, as in human cases, co-infections may be present and that infections may be asymptomatic [62]. Moreover, the higher infection risk in Thoroughbred could suggest an effect of the host genetics on infection susceptibility or of sexual transmission as in humans [37,63]. These findings confirm horses as a good model in comparative oncology in relation to PV infections.

## 5. Conclusions

This paper provides for the first time the genoprevalence of EcPV9 in Italian horses. Our results suggest four important conclusions: 1—in horses, as in humans, many infections are asymptomatic and probably resolve spontaneously; 2—Thoroughbred are more susceptible to the infection and show an OR of 26.4 to be positive with respect to other breeds used as a comparison variable; 3—in horses, as in humans, sexual transmission is a plausible model; 4—a very high genetic conservation of EcPV9 compared to other EcPVs was observed.

## Figures and Tables

**Table 2:** List of the EcPV isolates and their related accession numbers included in a house database used for NGS data analysis.

EcPV Type	Positive
Equus caballus papillomavirus type 1	AF498323.1
Equus caballus papillomavirus type 1	NC_003748.1
Equus caballus papillomavirus type 1 strain 150904	MF288893.1
Equus caballus papillomavirus type 1 isolate G2	MN164462.1
Equine papillomavirus 2	EU503122.1
Equine papillomavirus 2	NC_012123.1
Equine papillomavirus 2 isolate Zurich 2009	HM461973.1
Equus caballus papillomavirus 2 isolate XJ-ks1391	MW410986.1
Equine papillomavirus 3	NC_017862.1
Equine papillomavirus 3 isolate Haflinger	GU384895.1
Equus ferus caballus papillomavirus type 4	NC_020085.1
Equus ferus caballus papillomavirus type 4	JQ031032.1
Equus ferus caballus papillomavirus type 5	JQ031033.1
Equus ferus caballus papillomavirus type 5	NC_020084.1
Equine papillomavirus type 6	JQ965698.1
Equine papillomavirus type 6	NC_020500.1
Equus ferus caballus papillomavirus type 7	JX035935.1
Equus ferus caballus papillomavirus type 7	NC_020501.1
Equus caballus papillomavirus type 8 strain NCSU	KU963288.1
Equus caballus papillomavirus type 8 strain NCSU	NC_031756.1
Equus caballus papillomavirus 9 strain SW	MN117918.1

**Table 3:** EcPV9 genoprevalence detected in English Thoroughbred and Shire.

	Positive	%	Negative	%	Total
English Thoroughbred	10	13.9%	62	86.1%	72
Shire	1	25%	3	75%	4

**Table 4:** EcPV9 genoprevalence in the different age groups. The age of two horses was unknown.

	<6 yy	6–<9 yy	9–<13 yy	≥13 yy
Positive	0	2	6	1
Negative	41	32	56	63
Total	41	34	62	64

**Table 5:** EcPV9 genoprevalence on the basis of mares breed.

	Positive	%	Negative	%	Total
English Thoroughbred	10	16.1	52	83.9	62
Shire	1	25.0	3	75.0	4

## Data Availability

The original contributions presented in the study are included in the article/Supplementary Material. The raw reads generated in this study are available through the corresponding author upon request.

## Supplementary Materials

The following supporting information can be downloaded at: https://www.mdpi.com/article/10.3390/v14092050/s1, Figure S1: Map of Italy showing places of origin of horses positive for EcPV9-*L1*; Figure S2: Raw reads aligned to the reconstructed EcPV2 or EcPV9 genome and visualized through the Integrative Genome Viewer (IGV); Figure S3: Phylogenetic relationship between EcPV isolates obtained by an alignment of the completely reconstructed genomes of EcPV. The node labels indicate the bootstraps value supporting the clusterization, and the tree has been rooted on the EcVP1 group. When no isolate name is available, the accession number is reported. Figure S4: Alignment of ID2396_13-13 and ID2396_14-14 to the SW reference strain; Table S1: Samples positive for the detection of EcPV9-*L1* and EcPV2-*L1* by real-time PCR; Table S2: Follow-up of horses positive for EcPV9 and EcPV2; Table S3: NGS data analysis summary; Table S4: Evaluation of EcPV9 presence in the assembled contigs obtained through Blast alignments.
