# Supplementary material for: Equus caballus Papillomavirus Type-9 (EcPV9): First Detection in Asymptomatic Italian Horses
Source: Viruses. 2022 Sep 15;14(9):2050. doi: 10.3390/v14092050 (PMC9504741; doi:10.3390/v14092050)

Figure S2: Raw reads aligned to the reconstructed EcPV2 or EcPV9 genome and visualized through the Integrative Genome Viewer (IGV)

a) ID2396 2-2 raw reads aligned to the reconstructed EcPV2

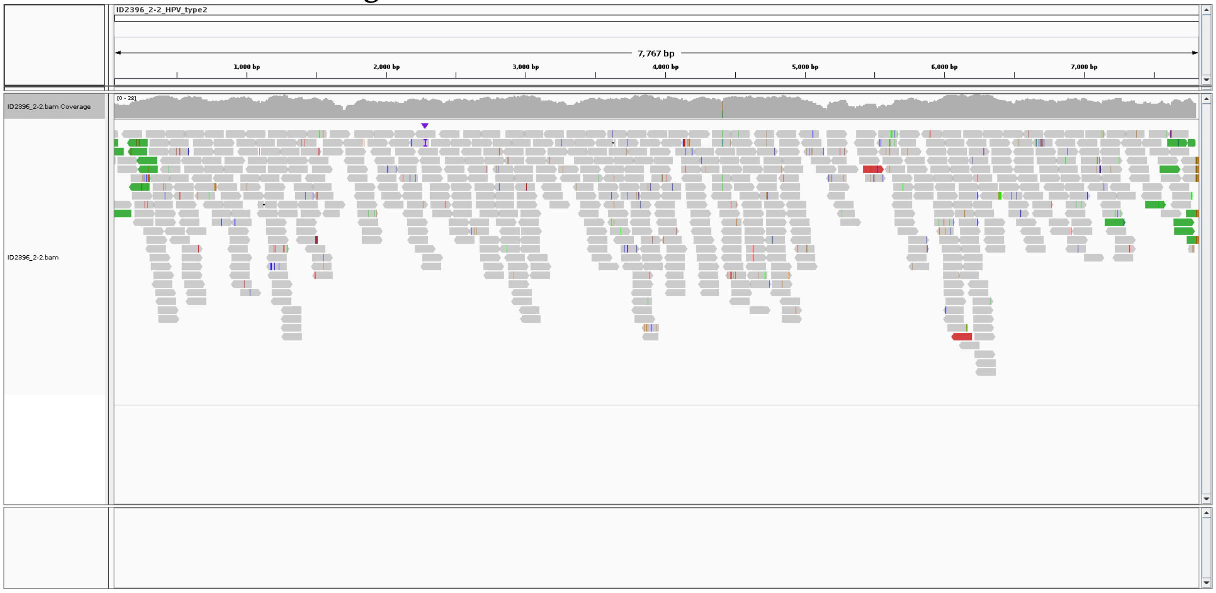

b) ID2396 3-3 raw reads aligned to the reconstructed EcPV2

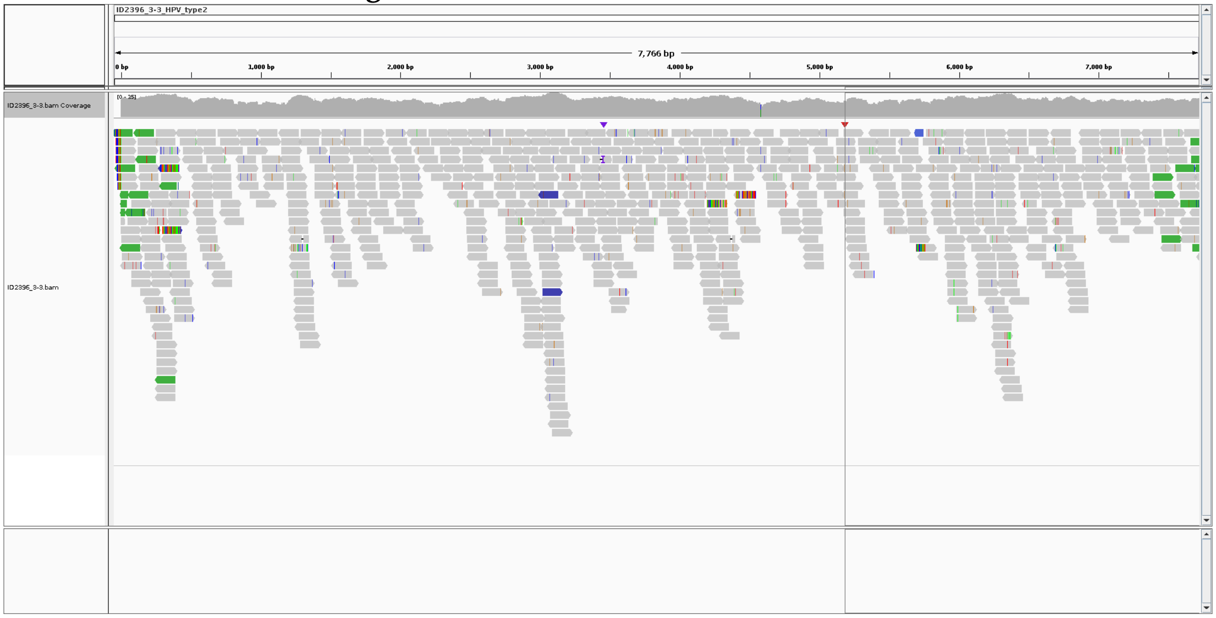

c) ID2396 5-5 raw reads aligned to the reconstructed EcPV2

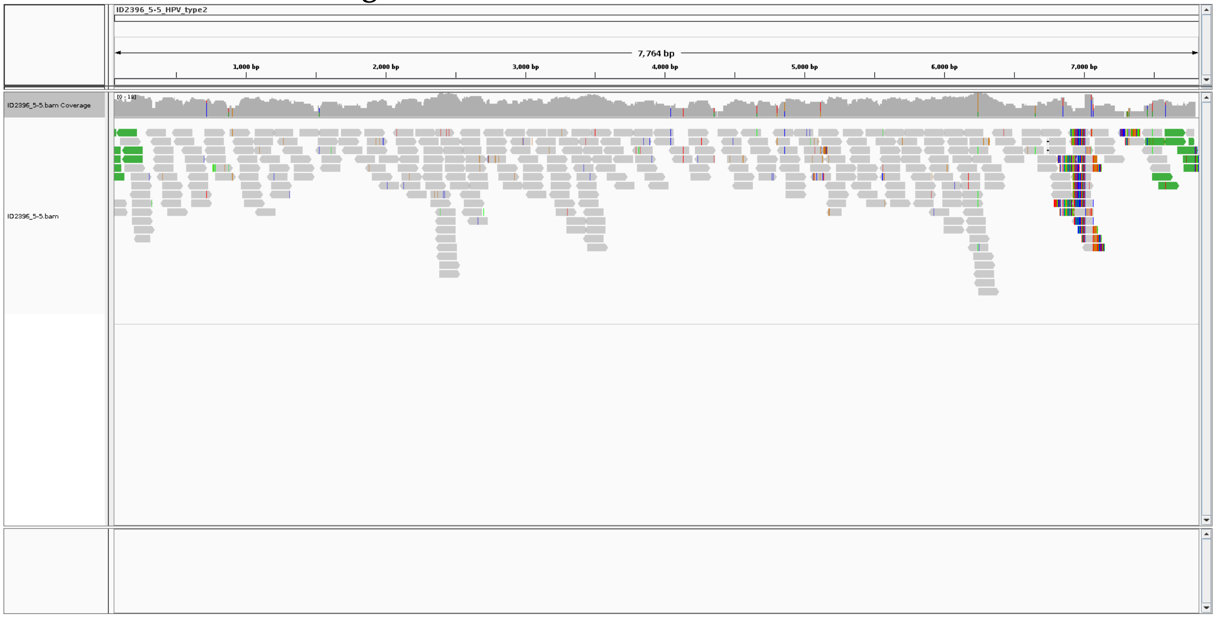

d) ID2396 8-8 raw reads aligned to the reconstructed EcPV2

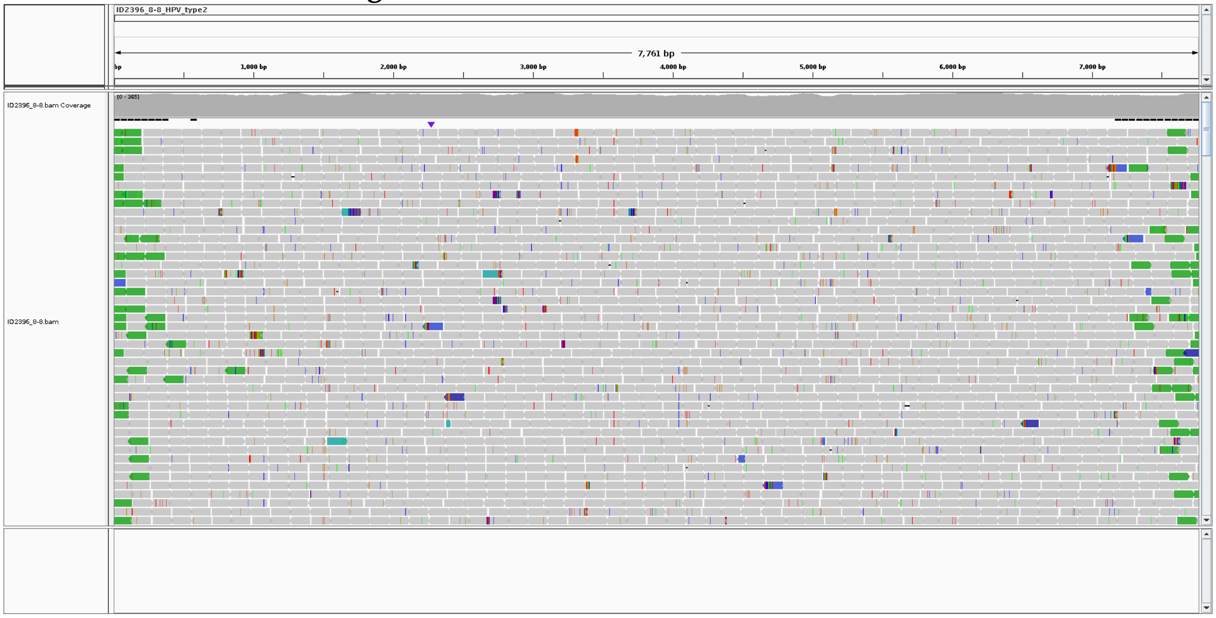

e) ID2396 13-13 raw reads aligned to the reconstructed EcPV9

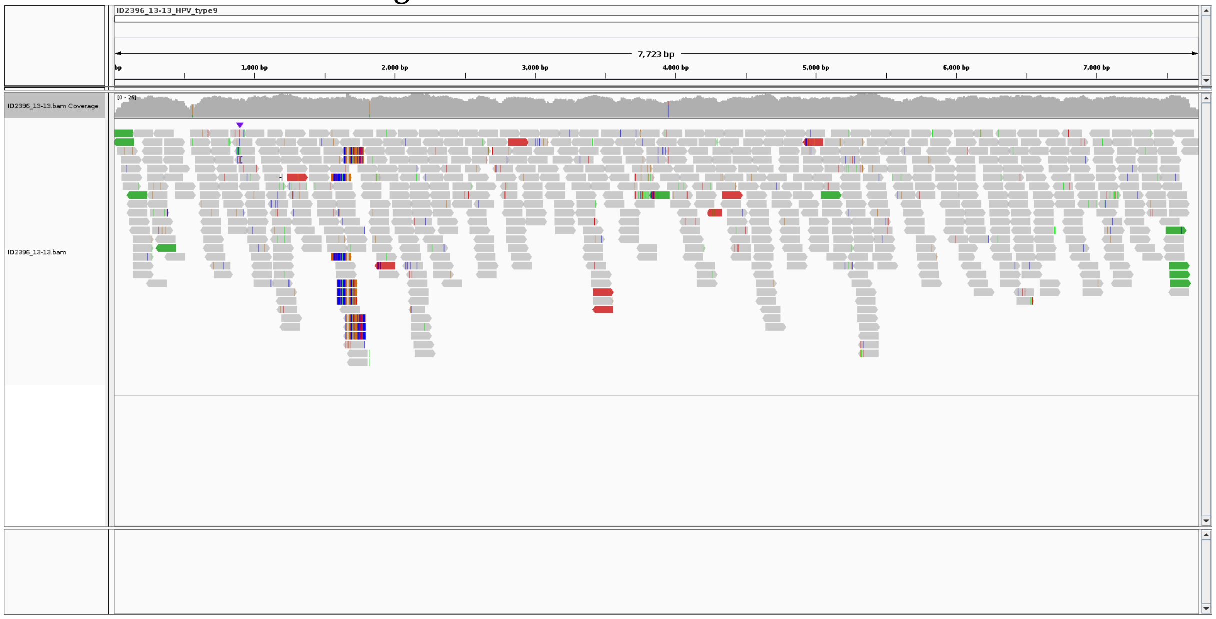

f) ID2396 14-14 raw reads aligned to the reconstructed EcPV9

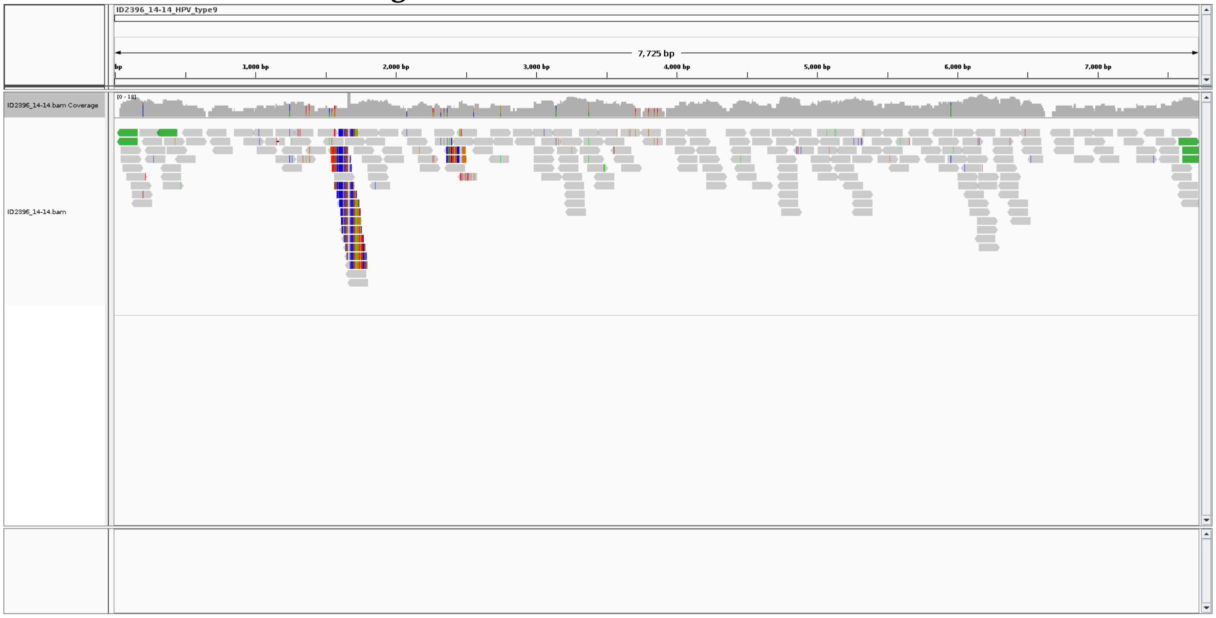

Supplement: Supplementary file 1 [file viruses-14-02050-s001.zip › supplementary/Figure S2.pdf]
