# Supplementary material for: Equus caballus Papillomavirus Type-9 (EcPV9): First Detection in Asymptomatic Italian Horses
Source: Viruses. 2022 Sep 15;14(9):2050. doi: 10.3390/v14092050 (PMC9504741; doi:10.3390/v14092050)
Supplement: Supplementary file 1 [file viruses-14-02050-s001.zip › supplementary/Table S1.pdf]

Table S1: Samples positive for the detection of EcPV9-L1 and EcPV2-L1 by Real Time PCR. Breed, age, sex and origin of sampled animals are reported in the table

ID	EcPV9-L1 Cq ± 1SD	EcPV2-L1 Cq ± 1SD	Breed	Age	Sex	Origin
ID71	31.59±3.33	23.8±1.0	Shire	10.0	F	Pralormo, Piedmont
ID18A	35.76±0.34	>38.00	English thoroughbred	N.A.	F	Vigone, Piedmont
ID143	35.72±0.65	>38.00	English thoroughbred	10.0	F	Pisa, Tuscany
ID151	35.42±0.66	>38.00	English thoroughbred	8.0	F	Vigone, Piedmont
ID107	28.79±0.74	32.4±0.2	English thoroughbred	9.0	F	Vigone, Piedmont
ID134	29.20±0.17	26.9±1.5	English thoroughbred	9.0	F	Faenza, Emilia Romagna
ID149	33.62±0.54	32.7±1.2	English thoroughbred	15.0	F	Umbertide, Umbria
ID163	33.60±0.56	35.3±0.6	English thoroughbred	8.0	F	Umbertide, Umbria
ID166	30.01±0.27	16.5±0.5	English thoroughbred	11.0	F	Noceto, Emilia Romagna
ID10A	32.87±0.36	33.59±1.84	English thoroughbred	11.0	F	Pralormo, Piedmont
ID75	27.85±0.22	>38.00	English thoroughbred	21.0	F	Siracusa, Sicily
