# Supplementary material for: Equus caballus Papillomavirus Type-9 (EcPV9): First Detection in Asymptomatic Italian Horses
Source: Viruses. 2022 Sep 15;14(9):2050. doi: 10.3390/v14092050 (PMC9504741; doi:10.3390/v14092050)
Supplement: Supplementary file 1 [file viruses-14-02050-s001.zip › supplementary/Table S2.pdf]

Table S2: Follow-up of horses positive for EcPV9 and EcPV2

ID	EcPV9-L1	EcPV2-L1	Follow up
ID71	+	+	Not pregnant after insemination
ID18A	+	-	Death following laminitis postpartum
ID143-17	+	-	Eutocic delivery
ID151-2	+	-	Occurrence of endometrial cysts; death following colic syndrome
ID107	+	+	Eutocic delivery
ID134	+	+	Occurrence of embryonal resorption
ID149	+	+	Occurrence of embryonal resorption
ID163-17	+	+	Occurrence of abortion
ID166-17	+	+	Eutocic delivery
ID10A	+	+	Occurrence of embryonal resorption
ID75	+	-	Not pregnant after insemination
