# Supplementary material for: Equus caballus Papillomavirus Type-9 (EcPV9): First Detection in Asymptomatic Italian Horses
Source: Viruses. 2022 Sep 15;14(9):2050. doi: 10.3390/v14092050 (PMC9504741; doi:10.3390/v14092050)
Supplement: Supplementary file 1 [file viruses-14-02050-s001.zip › supplementary/Table S3.pdf]

Table S3: NGS data analysis summary

Sample Name	Geographic origin	Total raw reads	Total trimmed reads	Total filtered reads	Coverage	Depth coverage
ID2396_2-2	Pralormo, Piemonte	25.677.870	18.960.896	512.666	100% EcPV2	12,11x
ID2396_3-3	Pralormo, Piemonte	27.288.860	19.722.286	547.602	100% EcPV2	13,4x
ID2396_5-5	Umbertide, Umbria	27.961.200	21.785.468	384.060	97.96% EcPV2	6,04x
ID2396_8-8	Noceto, Emilia Romagna	25.021.288	17.491.538	605.984	100% EcPV2	211,76x
ID2396_13-13	Siracusa, Sicilia	23.565.674	17.322.080	352.554	99.9% EcPV9	12.91x
ID2396_14-14	Vigone, Piemonte	21.529.102	14.806.828	203.626	98.13% EcPV9	4.14x
