# Supplementary figures and images for: Equus caballus Papillomavirus Type-9 (EcPV9): First Detection in Asymptomatic Italian Horses
Source: Viruses. 2022 Sep 15;14(9):2050. doi: 10.3390/v14092050 (PMC9504741; doi:10.3390/v14092050)

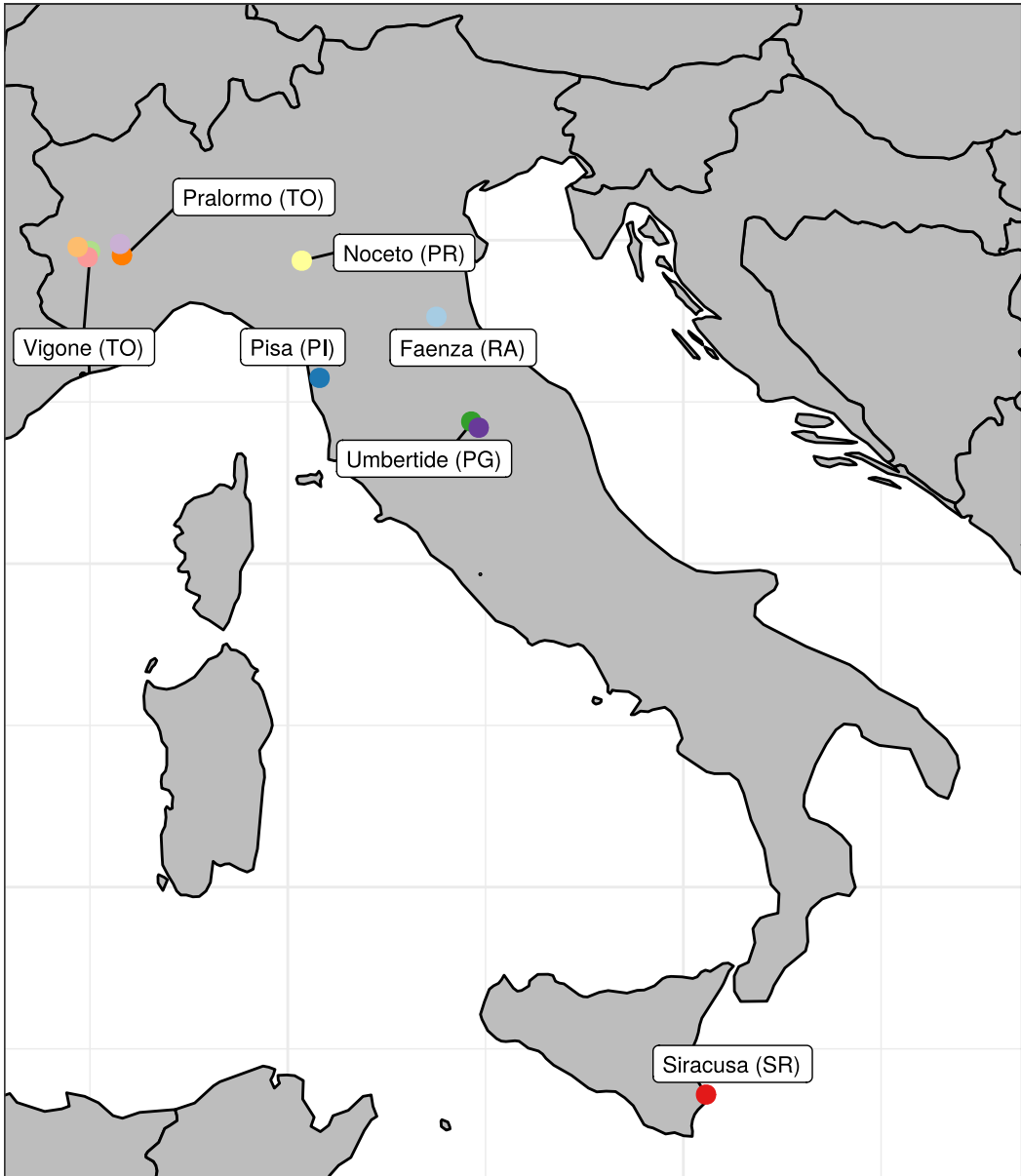

Supplement: Supplementary file 1 [file viruses-14-02050-s001.zip › supplementary/Figure S1.pdf]

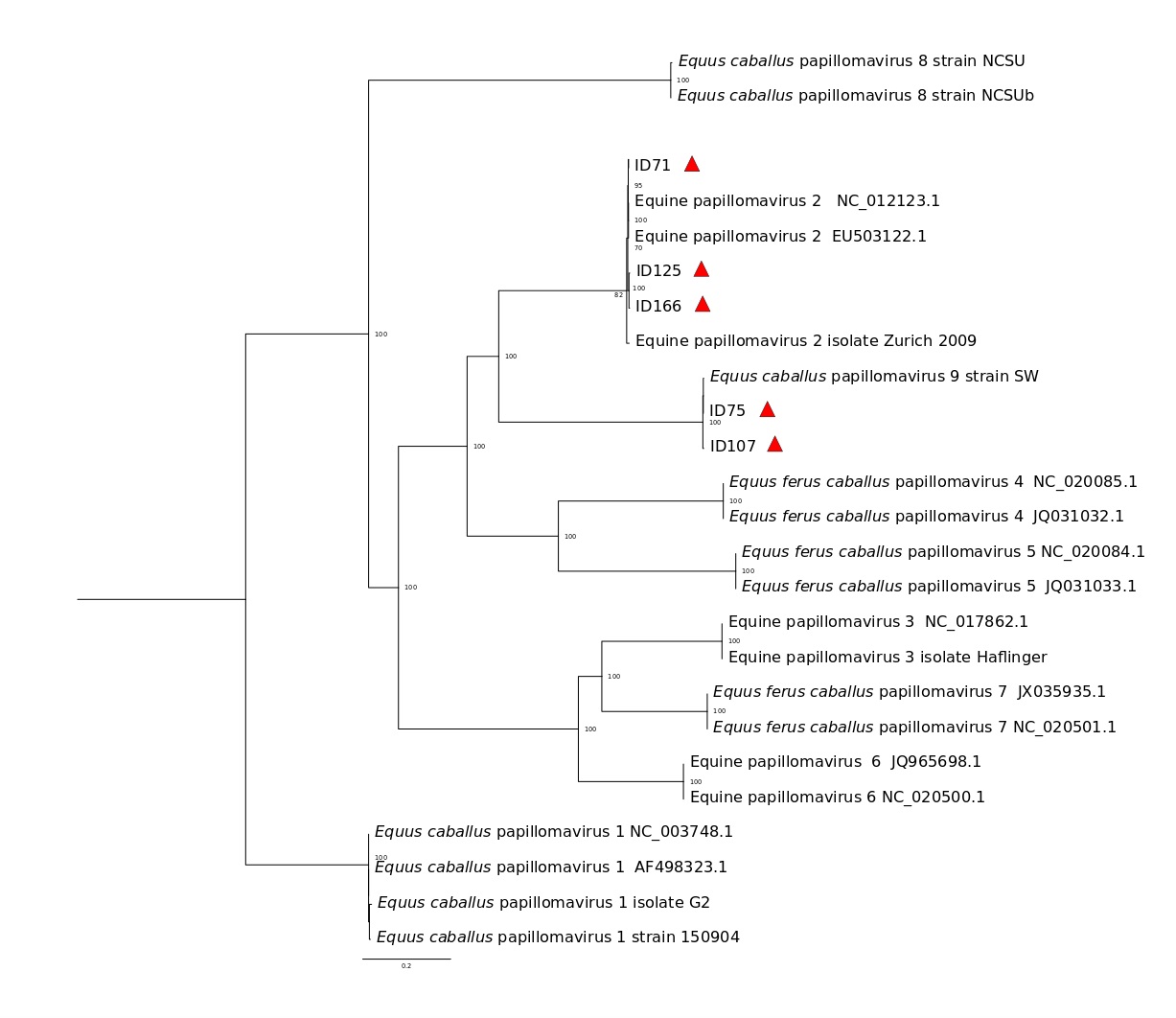

Supplement: Supplementary file 1 [file viruses-14-02050-s001.zip › supplementary/Figure S3.jpg]

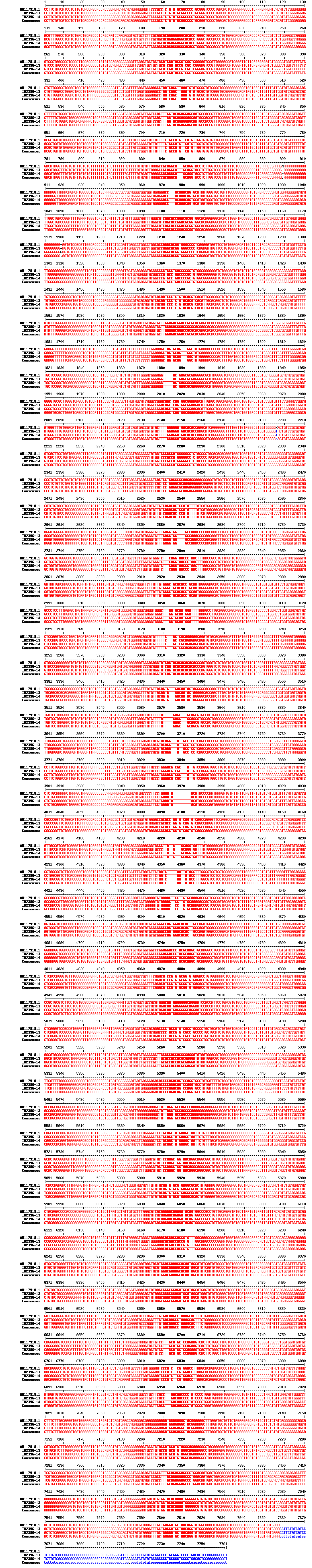

Supplement: Supplementary file 1 [file viruses-14-02050-s001.zip › supplementary/Figure S4.gif]
